# Effect of Channel Thickness on Amorphous In─Ga─Zn─O‐Thin‐Film Transistors

**DOI:** 10.1002/smsc.70432

**Published:** 2026-11-03

**Authors:** Hanjun Cho, Hiroshi Okanishi, Masato Sasase, Masatake Tsuji, Hideo Hosono

**Affiliations:** ^1^ MDX Research Center for Element Strategy International Research Frontiers Initiative Institute of Science Tokyo Yokohama Japan; ^2^ National Institute for Materials Science Tsukuba Ibaraki Japan

**Keywords:** amorphous oxide semiconductor, defect, thin‐film transistor

## Abstract

Thin‐film transistors based on amorphous oxide semiconductors demand thinner channels for next‐generation display and memory applications. However, channel‐thickness scaling causes a positive threshold‐voltage shift and degrades field‐effect mobility. Here, we report film densification in sputter‐deposited, hydrogen‐free amorphous indium–gallium–zinc oxide as the channel thickness decreases, with the film density exceeding that of single‐crystal InGaZnO_4_. Ab initio molecular dynamics simulations reveal a change in polyhedral connectivity from corner‐sharing to edge‐sharing. Fourier‐transform infrared spectroscopy and chemical etch‐rate measurements support this structural change, showing a shift of the M─O vibrational band peak to lower wavenumber and an increased etch rate. We propose that the resulting reduction in the M─O─M bond angle induces strain. In the proposed model, this strained local structure can enhance the formation of interstitial oxygen and lead to the formation of an oxygen Frenkel defect pair. This defect pair deepens the donor level and broadens the tail states below the conduction‐band minimum, thereby degrading thin‐film transistor performance. The degradation is suppressed in the In‐rich composition. This result may be understood in terms of the stabilization of interstitial oxygen as an electrically inactive peroxide species identified by the experimental results.

## Graphical abstract

We propose that densification in thinner a‐IGZO channels changes the local polyhedral connectivity from corner‐sharing to edge‐sharing, reducing the M─O─M bond angle and inducing M─O─M bond strain, which may promote oxygen Frenkel‐pair formation. These structural changes deepen the donor level, broaden unoccupied tail states, and degrade TFT performance, whereas a higher In/Ga ratio stabilizes interstitial oxygen and mitigates the degradation.

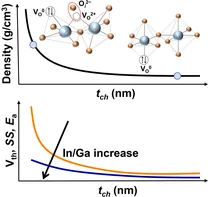

## 1 Introduction

Amorphous oxide semiconductors (AOSs) offer significant advantages owing to their low‐temperature processability, uniform thin‐film formation, and the ability to tailor their chemical composition and properties [1, 2, 3]. Among them, amorphous In─Ga─Zn─O (a‐IGZO) has become a de facto standard channel material for thin‐film transistors (TFTs) in display backplanes because of its well‐balanced performances (mobility, stability, and easy formation including sputter) in the amorphous state and compatibility with large‐area fabrication [4, 5, 6, 7]. Recently, reducing the channel thickness (*t*_ch_) has attracted increasing interest, driven by the demand for current‐driven organic light‐emitting diodes and next‐generation capacitor‐free dynamic random‐access memory applications, with the latter requiring nanometer‐thick a‐IGZO channels for continued device scaling and vertical integration [8, 9, 10, 11, 12, 13, 14, 15]. As the channel length is reduced, decreasing the channel thickness (*t*_ch_) is important for suppressing short channel effects and maintaining gate electrostatic control [15, 16, 17]. Despite this technological demand, controlling the electrical properties of a‐IGZO becomes significantly difficult as the *t*_ch_ decreases. This is because surface (back channel) and interface regions have a large impact on the carrier transport. Severe degradation in field‐effect mobility and electrical stability becomes particularly evident when the channel thickness is reduced below approximately 10 nm, limiting the application of a‐IGZO TFTs in highly scaled devices. Previous studies have attributed this thickness‐dependent degradation to channel depletion caused by back‐channel acceptor‐like traps, reduced doping efficiency, structural disorder, and deep electron traps associated with a defective near‐surface region [15, 18, 19, 20, 21, 22, 23]. Since these mechanisms are primarily regarded as surface‐related defects, back‐channel passivation has been employed. However, the persistence of the degradation after passivation suggests that its dominant origin lies within the film rather than solely at the surface. Therefore, understanding the bulk properties of ultrathin a‐IGZO films is essential for identifying the origin of the thickness‐dependent degradation. In the previous work, we reported a severe threshold voltage (*V*_th_) instability in very thin, hydrogen‐free a‐IGZO and proposed that donor‐level deepening originated from donor–acceptor interactions involving oxygen Frenkel defects [18]. In a separate study, the *μ*_FE_ degradation in very thin a‐IGZO was attributed to broadened conduction‐band tail states in a near‐surface region [23]. These studies reported the changes in electrical properties of very thin a‐IGZO, motivating further investigation of their structural origin. Understanding this structural origin is important for clarifying how the local structure of very thin a‐IGZO is related to defect formation. This article focuses on this point by employing several experimental methods and ab initio molecular dynamics (AIMD) simulation.

To isolate intrinsic thickness‐dependent behavior from hydrogen‐related effects, we prepared hydrogen‐free a‐IGZO films by ultra‐high‐vacuum sputtering. This is because hydrogen can act as a shallow donor and passivate oxygen‐related defects. Thus, the present results represent the intrinsic behavior of sputtered hydrogen‐free a‐IGZO when the thickness becomes thinner. Clarifying this intrinsic behavior provides a baseline for distinguishing fabrication‐induced effects in ALD‐grown and complex multilayer oxide devices with very thin thickness. We examined the thickness dependence of the film density and found that the density increases sharply, exceeding that of single‐crystal InGaZnO_4_ (c‐IGZO; 6.4 g/cm^3^) [24], when the film thickness (*t*_ch_) became below ~10 nm. The resulting densified a‐IGZO films suggest that they have a different local connectivity of the MO_*x*_ polyhedral from the conventional ones. To examine how this densification affects the local structure and electronic states, we investigated the M─O─M bond angle distribution and local polyhedral connectivity. These densification‐induced structural changes may be regarded as physical disorder in the amorphous network. In this article, we report (1) the densification of a‐IGZO films, (2) a model for the structural origin of the resulting electrical performance degradation supported by experimental and computational evidence, and (3) a composition‐based approach to mitigating this degradation. Based on this understanding, we show the In/Ga ratio as a practical design parameter for controlling the intrinsic electrical properties of ultrathin AOSs.

## 2 Densification and M─O─M Bond‐Angle Distortion in Very Thin a‐IGZO

To investigate the structural changes underlying the thickness‐dependent degradation, we measured the film density of a‐IGZO111 thin films by X‐ray reflectivity (XRR). Figure S1 shows the XRR profiles of the films with thicknesses of 5.1 and 28.3 nm. The extracted density increases from 6.10 to 6.78 g/cm^3^ as the thickness decreases from 28.3 to 5.1 nm. Figure 1a summarizes the film density as a function of thickness. The density remains near the bulk value of 6.10 g/cm^3^ above 20 nm but increases significantly below ~15 nm. In particular, the density of films thinner than 10 nm exceeds that of single‐crystal IGZO (~6.4 g/cm^3^) [24].

**FIGURE 1:**
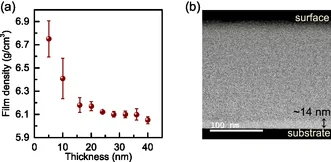
Increase in film density of a‐In_0.33_Ga_0.33_Zn_0.33_O_*x*_ (a‐IGZO111) with decreasing *t*_ch_. (a) Film density as a function of *t*_ch_, showing a significant increase below ~15 nm. Each data point represents the mean obtained from three independently prepared samples, and the error bars indicate the standard deviation. (b) Cross‐sectional low‐angle annular dark‐field scanning transmission electron microscopy (LAADF‐STEM) image of the interface region.

To examine the cross‐sectional microstructure, scanning transmission electron microscopy (STEM) observations were performed. Figure 1b shows a low‐angle annular dark‐field (LAADF)‐STEM image with a contrast variation extending ~14 nm from the substrate interface. No voids were observed in the present films. In addition, the corresponding high‐angle annular dark‐field and bright‐field STEM images show no comparable contrast variation between the near‐interface and bulk regions, as shown in Figure S2. The diffuse ring spacing decreased from 3.1 Å in the bulk region to 3.0 Å in the near‐interface region. This decrease may arise from changes in short‐range order or local strain (see Figure S2e). Further, energy‐dispersive X‐ray spectroscopy in TEM (TEM–EDS) and electron probe microanalysis revealed no detectable change in chemical composition with film thickness (Figure S2 and Table S1). These results indicate that the LAADF contrast reflects local strain or structural disorder rather than a discrete high‐density layer or compositional difference. It is generally reported that postannealing of a‐IGZO induces structural relaxation, with the density increasing by only 1%–3% as a result of this macroscopic relaxation [25, 26]. In contrast, the present films exhibit substantially larger densification than that expected from such thermal relaxation, indicating that a distinct microstructural change occurs in very thin films. To identify the origin of this microstructural change, we examined the local bonding structure of the films.

To examine how the densification affects the local bonding structure, the M─O vibrational states of the films were measured by Fourier‐transform infrared (FTIR) spectroscopy. Figure 2a shows the FTIR absorption spectra for thicknesses from 5 to 160 nm. The absorption coefficients (*α*) were estimated from the equation *α *= −ln[*T*/(1 − *R*)]/*d*, where *T* and *R* are the transmittance and reflectance, and *d* is the film thickness. The M–O vibrational band peak position near 415 cm^−1^ is assigned to the vibrational modes of the M (In, Ga, and Zn)─O bonds constituting the a‐IGZO network [27]. Figure 2b shows that the M─O vibrational band peak position shifts from 421 to 406 cm^−1^ as the thickness decreases from 160 to 5 nm. The observed shift to lower wavenumber suggests a decrease in the vibrational force constant of M─O network, which may result from decreased M─O─M bond angle. In the ultrathin film region, dense packing of the amorphous network would distort the M─O─M bond angles and shift the local polyhedral connectivity from corner‐sharing to edge‐sharing. These changes can account for the observed shift to lower wavenumber in Figure 2a,b. Similarly, a lower wavenumber shift was also reported for ion‐implanted silica glass, in which the bombardment‐induced volume compaction (~10%) reduced the Si─O─Si bridging bond angle by approximately 3°–9° and shifted the corresponding vibrational band to a lower wavenumber [28].

**FIGURE 2:**
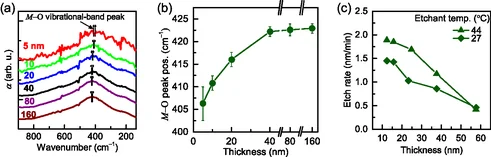
Fourier‐transform infrared (FTIR) spectra and wet‐etch rate as a function of film thickness. (a) FTIR absorption spectra of a‐IGZO111 films for thicknesses from 5 to 160 nm. (b) M─O vibrational‐band peak position as a function of thickness, showing a shift of approximately −15 cm^−1^ (from ~421 to ~406 cm^−1^) as the film becomes thinner. (c) Wet‐etch rate as a function of thickness.

To examine whether the strained M─O─M bonds resulting from these densification‐induced structural changes increase the chemical reactivity of the films, we measured their wet‐etch rates using a tetramethylammonium hydroxide (TMAH)‐based solution. Figure 2c shows the wet‐etch rate as a function of film thickness. The etch rate increases as the film thickness decreases. Conventionally, a denser oxide network is expected to etch more slowly owing to its more compact and chemically resistant structure. The opposite trend observed here indicates that the densified films contain strained M─O─M bonds, causing enhanced chemical reactivity. This behavior is also consistent with the stress corrosion model proposed for compacted a‐SiO_2_, in which the distorted network exhibits enhanced chemical reactivity because reducing the Si─O─Si bridging angle below its equilibrium value raises the potential energy of the bond much more steeply than an equivalent increase in the angle [28]. Such M─O─M bond strain in densified a‐IGZO can be regarded as physical disorder in the amorphous network.

The shift of the M─O vibrational band peak position to lower wavenumber and the increased etch rate suggest changes in the local bonding environment and enhanced chemical reactivity in the densified films. To identify the structural origin of these observations, we performed AIMD simulations using the densities obtained from XRR. Figure 3a shows the M─O─M bond angle distributions for film densities of 5.9, 6.3, and 6.7 g/cm^3^. Densification shifts the M─O─M connectivity from corner‐sharing toward edge‐sharing since a denser network requires the polyhedra to pack more closely. The density‐dependent changes in the overall M─O─M angle distribution over the density range from 5.9 to 6.9 g/cm^3^ and in the individual M─O─M components (In─O─In, In─O─Ga, In─O─Zn, Ga─O─Ga, Ga─O─Zn, and Zn─O─Zn) are provided in Figure S3. As the density increases, two notable changes are observed: (i) the low‐angle component peaking around 90° increases while the peak‐1 position remains nearly unchanged, and (ii) the higher‐angle component (peak‐2 in Figure 3a) decreases, with the peak‐2 fraction decreasing from 0.52 to 0.38 as the density increases (Table 1). The increase in the low‐angle component, with decreasing M─O─M angles toward ~90°, indicates a structural change in M─O polyhedral connectivity from corner‐sharing to edge‐sharing, as illustrated in Figure 3b. Previous molecular dynamics calculations for stoichiometric a‐InGaZnO_4_ showed that the effective coordination numbers of both metal and oxygen atoms increase with density [29]. In particular, the oxygen‐centered effective coordination number increased from 2.70 to 3.34 as the density increased from 5.17 to 7.05 g/cm^3^, supporting the interpretation that densification changes the local oxygen coordination environment.

**FIGURE 3:**
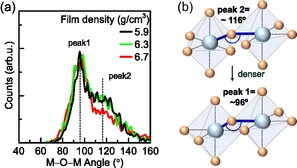
Structural change of the M─O─M network in a‐IGZO111 from ab initio molecular dynamics (AIMD) simulations. (a) M─O─M bond‐angle distributions for films with densities of 5.9, 6.3, and 6.7 g/cm^3^. As the density increases, a low‐angle component increases, whereas the intensity of the higher‐angle peak (peak 2) decreases. Note that only the In─O─In and In─O─Zn bonds are shown here, as these are the bonds that strongly contribute to carrier transport because they dominantly contribute to the conduction band bottom; the distributions for the other bonds are provided in the Supporting Information. (b) Schematic of the change in M–O polyhedral connectivity from corner‐sharing to edge‐sharing with densification.

**TABLE 1:** Experimental FTIR data and bond‐angle analysis obtained by AIMD. *A*_peak1_ and *A*_peak2_ denote the integrated areas of the peak‐1 and peak‐2 components, respectively.

(a) Experimental results			(b) AIMD results	
*t,* nm	*ρ*_exp._, g/cm^3^	M─O vibrational‐band peak pos., cm^−1^	*ρ*_cal._, g/cm^3^	*A*_peak2_/(*A*_peak1_ + *A*_peak2_)
40–80	6.10	421	5.9	0.52
20	6.17	417	6.3	0.52
10	6.41	410	6.7	0.38
5	6.78	406		

## 3 Donor‐Level Deepening and Tail‐State Broadening

To examine how these local structural changes and the resulting strain affect the electrical properties, temperature‐dependent Hall effect measurements were conducted. Figure 4a shows the Arrhenius plots of *n*_e_ for IGZO111 and IGZO631 at a thickness of 20 nm. The carrier concentration follows *n*_e _= *N*_D_·exp(−*E*_a_/*k*_B_ *T*), where *n*_e_, *N*_D_, *E*_a_, *k*_B_, and *T* denote the carrier concentration, the donor concentration, the activation energy, the Boltzmann constant, and the temperature, respectively. Increasing the Ga concentration is generally known to reduce *n*_e_ in a‐IGZO [1], but the present results show that this view is not fully correct. The temperature‐dependent Hall effect measurements determine both *N*_D_ and *E*_a_ and reveal that decreasing the In/Ga ratio does not necessarily reduce *N*_D_ but rather deepens the donor energy level. In fact, *N*_D_ is 9.5 × 10^19^ cm^−3^ for IGZO111, which is higher than the value of 5.5 × 10^17^ cm^−3^ for IGZO631, whereas *n*_e_ is lower in IGZO111. Because *n*_e_ depends exponentially on *E*_a_ but linearly on *N*_D_, the deepening of the donor level dominates over the increase in *N*_D_ [18]. Figure 4b summarizes *E*_a_ as a function of thickness. *E*_a_ increases as the film becomes thinner for both compositions, but the increase is much steeper for IGZO111 than for IGZO631. In our previous work, we proposed that this donor‐level deepening originates from donor–acceptor interactions involving oxygen Frenkel defects in thinner films [18]. In the very thin films, high concentrations of donor–acceptor pairs increase *E*_a_, suppressing *n*_e._

**FIGURE 4:**
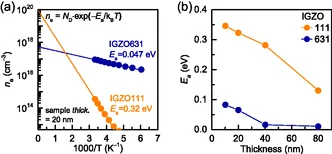
Donor level determined by temperature‐dependent Hall measurements. (a) Arrhenius plots of the carrier concentration (*n*_e_), *n*_e _= *N*_D_ · exp(−*E*_a_/*k*_B_ *T*), for a‐In_0.33_Ga_0.33_Zn_0.33_O_*x*_ (a‐IGZO111, In/Ga = 1) and a‐In_0.6_Ga_0.3_Zn_0.1_O_*x*_ (a‐IGZO631, In/Ga = 2) at a thickness of 20 nm, corresponding to activation energies of *E*_a_ ≈ 0.32 and 0.047 eV, respectively. (b) *E*_a_ as a function of thickness for both compositions; *E*_a_ increases as the films become thinner, much more steeply for IGZO111.

A possible alternative origin of the thickness‐dependent *E*_a_ is due to quantum confinement, which can widen the bandgap. We excluded this model because quantum confinement effects become significant only for *t*_ch_ below 5 nm [30]. In contrast, the films examined here are 10 nm or thicker, and the absorption spectra show no detectable change in the bandgap down to 10 nm (Figure S4), whereas *E*_a_ increases by approximately 0.2 eV as the thickness decreases from 80 to 10 nm in IGZO111.

The structural results obtained here provide a possible origin for the formation of these defects. Film densification changes the local M–O polyhedral connectivity from corner‐sharing toward edge‐sharing. Such a reduction of M─O─M bond angle induces a large strain, which in turn leads to enhanced oxygen interstitial formation. In the proposed model, the displacement of lattice oxygen to a nearby interstitial site forms O_i_^2−^ and leaves V_O_^2+^, that is, an oxygen Frenkel defect pair is formed. Oxygen Frenkel defects have been directly observed in amorphous SiO_2_ [31, 32, 33], and their possible existence in a‐IGZO has also been proposed. Recent first‐principles calculations further show that V_O_^2+^ can become thermodynamically stable depending on the local structure [34], supporting the V_O_^2+^ state assumed in the present model. The resulting donor–acceptor interactions deepen the V_O_^0^ donor level and suppress carrier generation.

To examine how the structural distortion affects device performance, we measured the transfer characteristics and *μ*_FE_ of bottom‐gate a‐IGZO TFTs as a function of *t*_ch_ for both compositions. The complete transfer characteristics and *μ*_FE_ curves for *t*_ch_ from 5 to 40 nm are provided in Figure S5. Figure 5a–c summarize *V*_th_, SS, and *μ*_FE_ as functions of *t*_ch_. Both compositions show a positive *V*_th_ shift, an increase in SS, and a reduction in *μ*_FE_ as the channel becomes thinner, but the degradation is more severe in IGZO111. Figure 5a,b show the thickness dependences of *V*_th_ and SS, respectively. For IGZO111, *V*_th_ increases from approximately 1.5–12 V, and SS increases from 0.25 to 0.9 V/dec as the thickness decreases from 40 to 5 nm. In contrast, for IGZO631, *V*_th_ changes only from 0.5 to 0.7 V, while SS remains near 0.2 V/dec over the same thickness range. Figure 5c shows that the initial *μ*_FE_ is consistently lower in IGZO111 than in IGZO631 and decreases more rapidly as the film becomes thinner. Device‐to‐device statistics for *V*_th_, SS, and *μ*_FE_ at *t*_ch_ = 5 and 40 nm are summarized in Table S2. The positive *V*_th_ shift, increase in SS, and reduction in the initial *μ*_FE_ suggest that unoccupied electron‐trapping states are distributed below the CBM and trap carriers under positive gate bias. The more severe degradation observed in IGZO111 indicates greater electron trapping in the Ga‐rich composition.

**FIGURE 5:**
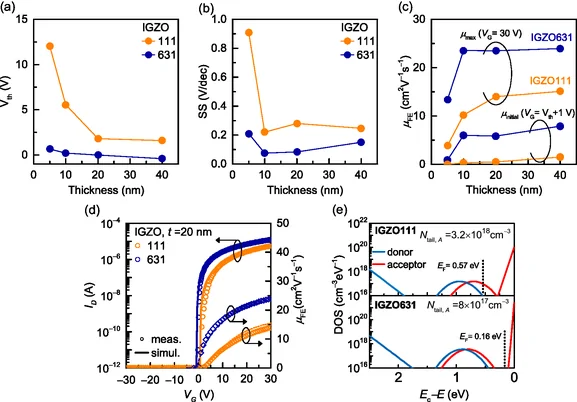
Composition‐dependent TFT degradation and bulk unoccupied tail states. (a) *V*_th_, (b) subthreshold swing (SS), and (c) initial and maximum field‐effect mobility (*μ*_FE_) as functions of *t*_ch_ for IGZO111 and IGZO631. The initial and maximum *μ*_FE_ values were evaluated at *V*_G _= *V*_th_ + 1 and 30 V, respectively. (d) Measured (symbols) and simulated (lines) transfer characteristics and *μ*_FE_ curves for 20‐nm‐thick IGZO111 and IGZO631. (e) Extracted bulk DOS distributions for IGZO111 (upper) and IGZO631 (lower), plotted relative to their respective CBMs. The vertical dashed lines indicate Fermi level (*E*_F_).

To quantify the unoccupied tail states responsible for the degradation, we reproduced the measured transfer characteristics by technology computer‐aided design (TCAD) analysis using a bilayer DOS model consisting of a defective surface region and a bulk region. This bilayer DOS model follows a previous model for sputter‐deposited a‐IGZO, in which a defective near‐surface layer formed during sputter deposition was identified by depth‐resolved hard X‐ray photoemission spectroscopy [23]. The comparison was performed at a thickness of 20 nm so that the contribution of the defective surface layer to carrier transport is negligibly small, and the composition‐dependent difference in the bulk region can be examined. Structural disorder in amorphous oxides can create fluctuations in the disorder potential that perturb the conduction‐band (CB) edge, leading to Anderson localization and broadening of the unoccupied tail states below the CBM [23]. Therefore, the electron‐trapping states were modeled as exponential unoccupied tail states rather than discrete Gaussian acceptor levels. The complete surface and bulk DOS distributions and fitting parameters are provided in Figure S6 and Tables S3 and S4. Figure 5d shows the measured and simulated transfer characteristics of 20‐nm thick IGZO111 and IGZO631. The data are reproduced by varying only the bulk acceptor‐like conduction‐band tail width (*W*_TA_), while the remaining parameters are kept identical between the two compositions. Figure 5e compares the extracted bulk density‐of‐states (DOS) distributions. The CB tail width *W*_TA_ is 32 meV for IGZO111 and 8 meV for IGZO631, making the IGZO111 value 4 times larger. The *W*_TA_ values reported for a‐IGZO range from 8 to 15 meV in TCAD analyses [23, 35, 36]. The value for IGZO631 lies within this range, whereas that for IGZO111 is significantly larger. The integrated conduction‐band tail‐state density (*N*_TA_) is 3.2 × 10^18^ cm^−3^ for IGZO111 and 8 × 10^17^ cm^−3^ for IGZO631. These results identify wider broadening and a higher density of bulk unoccupied tail states in IGZO111. Taken together, the Hall effect measurements and TCAD analysis identify two changes in the bulk DOS: deepening of the V_O_^0^ donor level and broadening of the unoccupied tail states below the CBM. We propose that these changes arise from densification‐induced structural disorder. The deepened donor level suppresses carrier generation, whereas the broadened tail states trap gate‐induced electrons and hinder the shift of *E*_F_ toward the mobility edge under positive gate bias. Our previous TCAD analysis showed that increasing *W*_TA_ from 0.01 to 0.03 eV at a fixed *N*_TA_ of 1 × 10^20^ cm^−3^ increased SS from 0.11 to 0.22 V/dec for a 30‐nm channel and from 0.20 to 0.69 V/dec for a 5‐nm channel [37]. These two effects account for the positive *V*_th_ shift, increase in SS, and reduction in the initial *μ*_FE_ observed in Figure 5.

## 4 Chemical Composition‐Dependent Stabilization of Interstitial Oxygen

The temperature‐dependent Hall effect measurements and TCAD analysis revealed substantial differences in the donor levels and unoccupied tail states of IGZO111 and IGZO631. This composition dependence suggests that the stabilization of the interstitial oxygen (O_i_) formed by the Frenkel‐type displacement differs between the Ga‐rich and In‐rich compositions. Accordingly, we investigated the O_i_ species and experimentally examined how its behavior differs with chemical composition. In Ga‐rich thin films, O_i_ may remain as an individual O_i_^2−^ species, whereas in In‐rich thin films, it may form an O─O bond as a reaction with lattice oxygen, as suggested by Density functional theory (DFT) calculations [38, 39, 40, 41]. To examine whether an In‐rich oxide can stabilize such O─O‐bonded oxygen species, we performed ESR measurements. For this comparison, IGZO613 (In_0.6_Ga_0.1_Zn_0.3_O_*x*_) was selected to examine the effect of the In/Ga ratio at a fixed Zn fraction. Figure 6 compares the ESR spectra of IGZO111 and In‐rich IGZO613. No O_2_^−^ signal was detected for IGZO111 (Figure 6a), whereas IGZO613 exhibited features at *g*_1_ ≈ 2.042–2.138, *g*_2_ = 2.004, and *g*_3_ = 1.991, which are characteristic of O_2_^−^ (Figure 6b). These results suggest that increasing the In/Ga ratio promotes O─O bond formation. The same tendency was observed in In_2_O_3_, used as an In‐rich compositional‐limit reference. In_2_O_3_ exhibited a feature at *g*_2_ = 2.009 and a low‐field shoulder corresponding to *g*_1_ ≈ 2.032–2.105 (Figure 6c), both of which are characteristic of O_2_^−^ [42]. The observation of O_2_^−^ in both IGZO613 and In_2_O_3_ supports the ability of In‐rich oxides to stabilize O─O‐bonded oxygen species. Since ESR detects only paramagnetic O_2_^−^ and not diamagnetic O_2_^2−^, these results show the ability to form an O─O bond in In‐rich thin films but do not directly determine the charge state of the O─O unit in IGZO631. Nevertheless, these results support the possibility that interstitial oxygen is stabilized through O─O bond formation in the In‐rich composition. Based on this ESR result and the calculated defect formation energies [38, 39, 40, 41], we propose the composition‐dependent models shown in Figure 6d,e.

**FIGURE 6:**
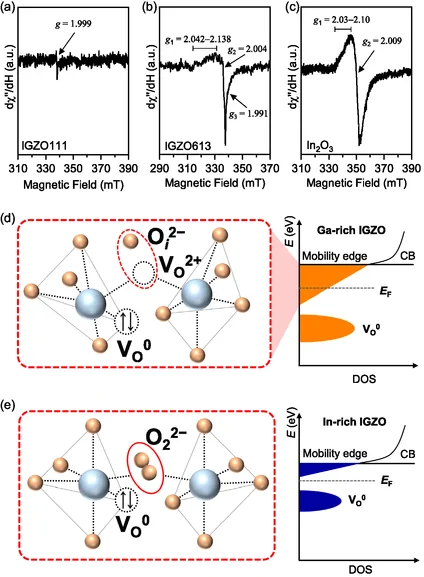
Composition‐dependent behavior of interstitial oxygen, corresponding DOS, and electron spin resonance (ESR) spectra. The ESR spectra in (a–c) were measured at 77 K. A 1000‐nm‐thick film was used for each sample to improve the signal‐to‐noise ratio. (a) ESR spectrum of IGZO111, showing no detectable O_2_^−^ signal. (b) ESR spectrum of a‐IGZO613; *g*_1_ ≈ 2.042–2.138, *g*_2_ = 2.004, and *g*_3_ = 1.991 are consistent with O_2_^−^. (c) ESR spectrum of a crystalline In_2_O_3_ thin film as an In‐rich compositional‐limit reference; *g*_1_ ≈ 2.032–2.105 and *g*_2_ = 2.009 are consistent with O_2_^−^. (d) Proposed oxygen Frenkel‐defect model for IGZO111 and corresponding DOS. (e) Proposed peroxide model for In‐rich IGZO and corresponding DOS.

In the Ga‐rich thin films (Figure 6d), a displaced lattice oxygen in IGZO111 remains as an O_i_^2−^ species localized at an interstitial site and leaves V_O_^2+^, forming an oxygen Frenkel defect pair of V_O_^2+^ and O_i_^2−^. V_O_^2+^ represents oxygen vacancies without electrons, whereas V_O_^0^ denotes oxygen vacancies with two electrons. In our previous work, the O 1s peak at ~531 eV was assigned to oxygens perturbed by interstitial O_i_^2−^ and V_O_ [18]. The M─O─M angle distortion identified here provides a possible structural origin for this perturbed local structure. The schematic DOS in Figure 6d shows a high concentration of deep V_O_^0^ donors, consistent with the larger *E*_a_ and *N*_D_ obtained from the Hall effect measurements (Figure 4a), together with broad CB tail states extending below the CBM. In the In‐rich samples (Figure 6e), O_i_ bonds with a lattice oxygen to form a peroxide O─O unit. In n‐type IGZO631, this O─O unit can trap an additional electron and become an electrically inactive peroxide anion (O_2_^−^ + e^−^ → O_2_^2−^). Compared with IGZO111, IGZO631 exhibits a shallower V_O_^0^ donor level, a lower V_O_^0^ donor concentration, and narrower unoccupied tail states below the CBM. In this model, O_i_^2−^ species in IGZO111 contribute to broadening of the bulk tail states, whereas formation of electrically inactive O_2_^2−^ in In‐rich IGZO631 suppresses this broadening. No detectable O_2_^−^ ESR signal was observed for IGZO631. Thus, the shallower donor level and narrower unoccupied tail states of IGZO631 suggest that Frenkel‐related defects are suppressed, while the concentration of O─O‐bonded oxygen species remains below the ESR detection limit. IGZO631 represents an intermediate composition between IGZO111 and IGZO613, maintaining stable TFT operation.

## 5 Conclusions

The primary points obtained are summarized as follows:

I. Very thin sputtered a‐IGZO films exhibit densification, with film densities exceeding the density of single‐crystal InGaZnO_4_. LAADF‐STEM indicates a locally strained and structurally disordered region extending ~14 nm from the interface.
II. AIMD simulations reveal that a‐IGZO densification leads to a decrease in the M─O─M bond angle and a change in local polyhedral connectivity from corner‐sharing to edge‐sharing. This structural change increases the strain in the M─O─M bonds, consistent with the shift of the M─O vibrational band peak to lower wavenumber and the increased etch rate.
III. Based on these structural results, we propose that densification reduces M─O─M bond angle, which induces strain in M─O─M bond. Such a strained bond may make the formation of oxygen Frenkel pair (V_O_^2+^ + O_i_^2−^) easier.
IV. Temperature‐dependent Hall effect measurements and TCAD analysis reveal deepening of the V_O_^0^ donor level and broadening of the unoccupied tail states below the CBM, respectively.
V. The deepened V_O_^0^ donor level suppresses carrier generation, while the broadened unoccupied CB tail states trap electrons under positive *V*_G_, causing a positive *V*_th_ shift, an increase in SS, and a reduction in the initial *μ*_FE_.
VI. In the In‐rich composition, O_i_ bonds with a lattice oxygen to form an electrically inactive O_2_^2−^ species, rather than remaining as an individual O_i_^2−^ species as in the Ga‐rich composition, as supported by ESR measurements and DFT calculations. Increasing the In/Ga ratio mitigates oxygen Frenkel defects by stabilizing interstitial oxygen as an electrically inactive peroxide species.

This study identifies densification‐induced structural distortion and proposes that it may promote the formation of oxygen Frenkel defects, providing a plausible origin for TFT performance degradation. These findings clarify the thickness‐ and chemical‐composition‐dependent behavior of sputtered hydrogen‐free a‐IGZO and provide a baseline for understanding oxide‐semiconductor behavior in next‐generation electronics.

## 6 Experimental Section

### 6.1 Thin‐Film Deposition

To compare the structural and electrical properties across chemical compositions, two compositions with different In/Ga ratios were prepared: a‐In_0.33_Ga_0.33_Zn_0.33_O_*x*_ (a‐IGZO111, In/Ga = 1) and a‐In_0.6_Ga_0.3_Zn_0.1_O_*x*_ (a‐IGZO631, In/Ga = 2), while a‐In_0.6_Ga_0.1_Zn_0.3_O_*x*_ (a‐IGZO613, In/Ga = 6) was prepared for ESR measurements to examine the effect of the In/Ga ratio at a fixed Zn fraction. To examine the intrinsic thickness dependence without the influence of hydrogen, the films were deposited by ultrahigh‐vacuum RF magnetron sputtering under cryo‐pumping. Hydrogen incorporated during deposition can act as a shallow donor and passivate oxygen‐related defects, obscuring the intrinsic behavior of a‐IGZO. The a‐IGZO films, ranging from 5 to 160 nm in thickness, were deposited by RF magnetron sputtering without intentional heating. The sputtering conditions were as follows: an RF power of 150 W, 3‐inch‐diameter target, a total pressure of 0.4 Pa, an oxygen partial‐pressure ratio of P_O2_ = O_2_/(Ar + O_2_) = 25%, and a base pressure of approximately 2 × 10^−7^ Pa achieved by cryo‐pumping. Thermal desorption spectroscopy showed no detectable H_2_ desorption from the ultra‐high‐vacuum‐sputtered films, corresponding to a hydrogen concentration below the detection limit of approximately 10^18^ cm^−3^. In contrast, conventionally sputtered films prepared at a base pressure of approximately 10^−4^ Pa contained approximately 10^20^ cm^−3^ hydrogen (Figure S7). After deposition, the samples were annealed at 400 °C for 1 h in ambient air.

### 6.2 TFT Device Fabrication

Bottom‐gate, top‐contact TFTs were fabricated for both compositions. P^++^‐silicon substrates with a 150 nm thermally grown SiO_2_ gate insulator were used. The a‐IGZO films, ranging from 5 to 40 nm in thickness, were deposited under the conditions described in Section 6.1, followed by the same postdeposition annealing. The TFT channels were patterned with a width of 300 μm and a length of 30 μm by photolithography (MA‐10, Mikasa). Mo (8 nm) and Au (30 nm) were sequentially deposited as the source and drain electrodes. The threshold voltage (*V*_th_) was determined as the *V*_GS_ at $I_{D}=\frac{W}{L}\times 50\;\text{pA}$, whereas the subthreshold swing (SS) was taken as $\mathrm{SS}=dV_{\mathrm{GS}}/d\log\;(I_{D})$, within the range of 10 pA < *I*_D_ < 100 pA. The field‐effect mobility (*µ*_FE_) value was calculated from $\mu_{\text{Linear}}=\frac{L}{WC_{\mathrm{OX}}\;V_{\mathrm{SD}}}g_{m_{\max}}$.

### 6.3 Characterization of Devices and Thin‐ Films

The film thickness and density were measured by grazing‐incidence XRR, (SmartLab, Rigaku). The cross‐sectional microstructure was examined by STEM, (JEM ARM200F, JEOL Ltd.). FTIR transmittance and reflectance spectra were collected from a‐IGZO111 films deposited on 200‐μm‐thick, double‐side‐polished, nondoped Si substrates using a VERTEX 70v spectrometer (Bruker) under vacuum at room temperature. The wet‐etch rate was evaluated by etching the films in a 1.6 wt% tetramethylammonium hydroxide (TMAH) solution at 27 and 44 °C; the film thickness was measured by XRR before and after etching, and the etch rate was calculated from the thickness difference divided by the etching time. The carrier transport properties were investigated by AC magnetic field Hall effect measurement (ResiTest8400, TOYO) with the van der Pauw configuration under an AC magnetic field of 0.4 T from 150 to 300 K in a helium atmosphere. The transfer characteristics and *µ*_FE_ of the TFTs were measured using a semiconductor parameter analyzer (B2912A, Keysight). O_2_^−^ species in a‐IGZO111, IGZO613, and In_2_O_3_ films were examined by electron spin resonance spectroscopy (ESR; Bruker EMX) at 77 K.

### 6.4 Ab Initio Molecular Dynamics Simulations

The amorphous In─Ga─Zn─O (a‐IGZO) structures were generated via AIMD liquid‐quench simulations using the Vienna Ab Initio Simulation Package [43, 44, 45, 46]. DFT calculations [47, 48] were performed utilizing the Perdew–Burke–Ernzerhof (PBE) exchange‐correlation functional within the projector augmented‐wave (PAW) method [49, 50, 51]. To systematically investigate the density dependence of the amorphous network, six different target densities were employed: 5.9, 6.1, 6.3, 6.5, 6.7, and 6.9 g/cm^3^. A stoichiometric crystalline IGZO (c‐IGZO) cell consisting of 336 atoms (In_48_Ga_48_Zn_48_O_192_) was used as the initial structure for each density. The liquid‐quench MD sequence was performed in the NVT ensemble with the Nosé‐Hoover thermostat using an integration time step of 2 fs, following a liquid‐quench protocol for amorphous oxides [52]. First, the initial structures were melted and equilibrated at 3000 K for 10 ps (5000 steps). The liquids were then cooled to 2500 K over 5 ps (2500 steps) at a cooling rate of 100 K/ps and subsequently quenched to 100 K over 12 ps (6000 steps) at a rate of 200 K/ps. During these melting and quenching stages, a plane‐wave energy cutoff of 400 eV and a single Γ‐point were employed. Following the quench, the structures were equilibrated at 300 K for 6 ps (3000 steps) with the same cutoff energy of 400 eV. Finally, a full structure optimization (zero‐temperature relaxation) was performed for each quenched structure to obtain the final static configurations of the a‐IGZO cells using an increased energy cutoff of 520 eV and a 4 × 4 × 2 k‐point grid.

### 6.5 TCAD Analysis

The density of states of the acceptor tail was extracted by reproducing the measured transfer characteristics using a TCAD simulator (Silvaco Victory). A bilayer DOS model consisting of a defective surface region and a bulk region was employed [23], and the acceptor tail parameters were adjusted to reproduce the data. The complete DOS parameters used for the surface and bulk regions of IGZO111 and IGZO631, including the acceptor‐ and donor‐like tail and Gaussian state distributions, are summarized in Table S4.

## Funding

This study was supported by the Japan Society for the Promotion of Science (JP26K17924), and Samsung Electronics Co., Ltd. (IO250530‐13006‐01).

## Conflicts of Interest

The authors declare no conflicts of interest.

## Supporting information

Supplementary Material

## Data Availability

The data that support the findings of this study are available from the corresponding author upon reasonable request.
