# Supplementary material for: Effect of Channel Thickness on Amorphous In─Ga─Zn─O‐Thin‐Film Transistors
Source: Small Sci. 2026 Nov 3;6(11):e70432. doi: 10.1002/smsc.70432 (PMC13729734; doi:10.1002/smsc.70432)
Supplement: Supplementary file 1 [file SMSC-6-e70432-s001.pdf]

Supporting Information

Effect of Channel Thickness on Amorphous IGZO-TFTs

Authors: *Hanjun Cho*[1], *Hiroshi Okanishi*[1], *Masato Sasase*[1], *Masatake Tsuji*[1*], *and Hideo Hosono*[1, 2*]

Table of contents

Figure S1. XRR profiles of a-IGZO111

Figure S2. Cross-sectional STEM, EDS, and electron diffraction analyses of a-IGZO111.

Figure S3. AIMD analysis of the *M*–O–*M* bond-angle distribution as a function of film density.

Figure S4. Tauc plots for (a) IGZO111 and (b) IGZO631 at thicknesses of 10, 20, 50, and 100 nm.

Figure S5. Complete transfer characteristics and field-effect mobility curves as functions of channel thickness.

Figure S6. Complete TCAD-extracted DOS distributions for IGZO111 and IGZO631.

Figure S7. Thermal desorption spectroscopy of hydrogen (m/z = 2, H_2) from a-IGZO films deposited by conventional and ultra-high-vacuum (UHV) sputtering.

Table S1. Electron probe microanalysis (EPMA) of the cation composition of a-IGZO111 films as a function of thickness.

Table S2. Device-to-device variation in V_{th}, *SS*, and μ_{FE} for IGZO111 and IGZO631 TFTs with channel thicknesses of 5 and 40 nm.

Table S3. Density-of-states (DOS) parameters used in the device simulations for IGZO111 and IGZO631, extracted separately for the surface and bulk regions.

Table S4. CB tail widths and integrated tail-state densities for IGZO111 and IGZO631.

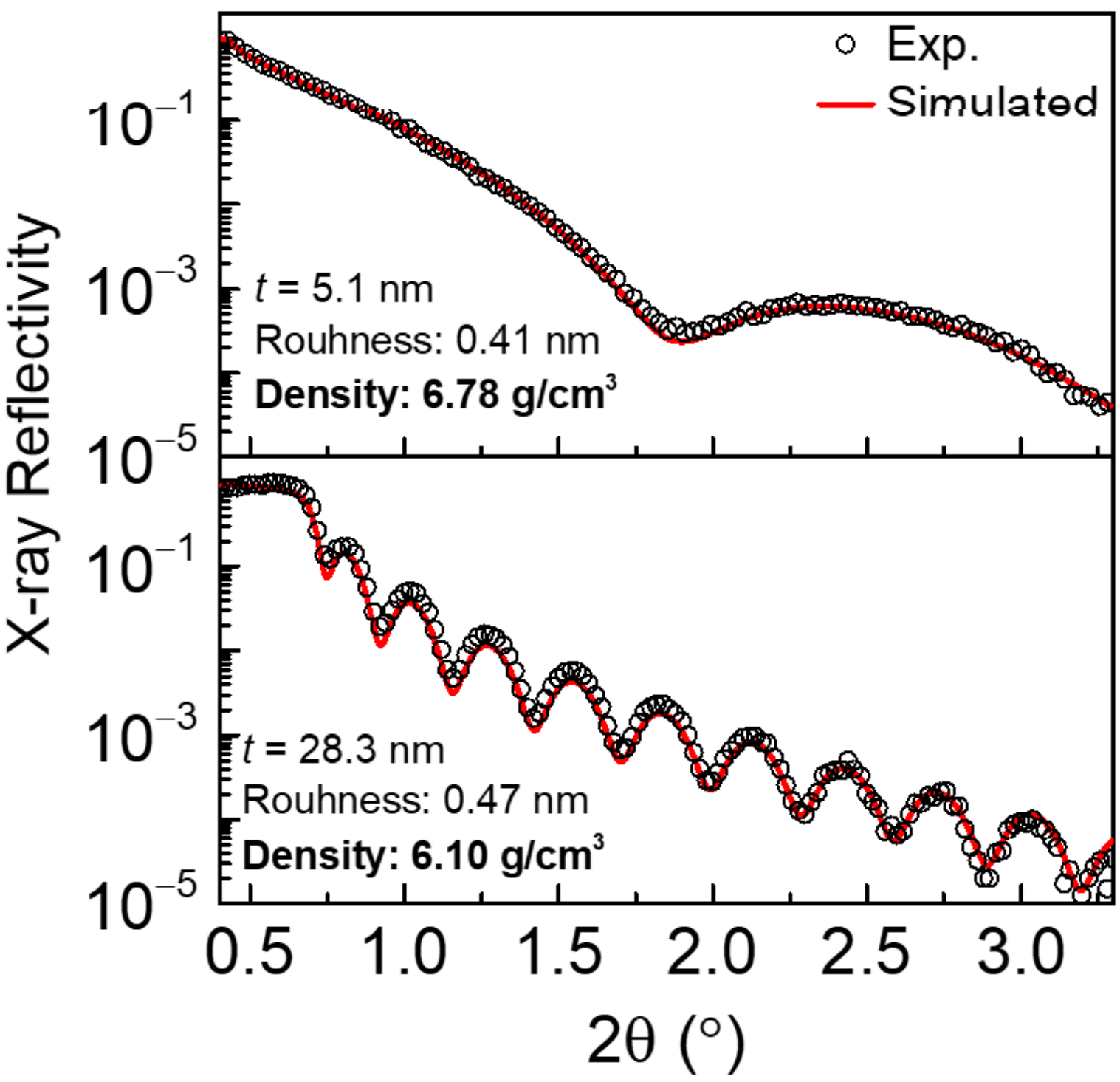

Figure S1. X-ray reflectivity (XRR) profiles (open circles: experimental; red lines: simulated) of a-IGZO111 films with thicknesses of 5.1 and 28.3 nm, corresponding to densities of 6.78 and 6.10 g/cm3, respectively.

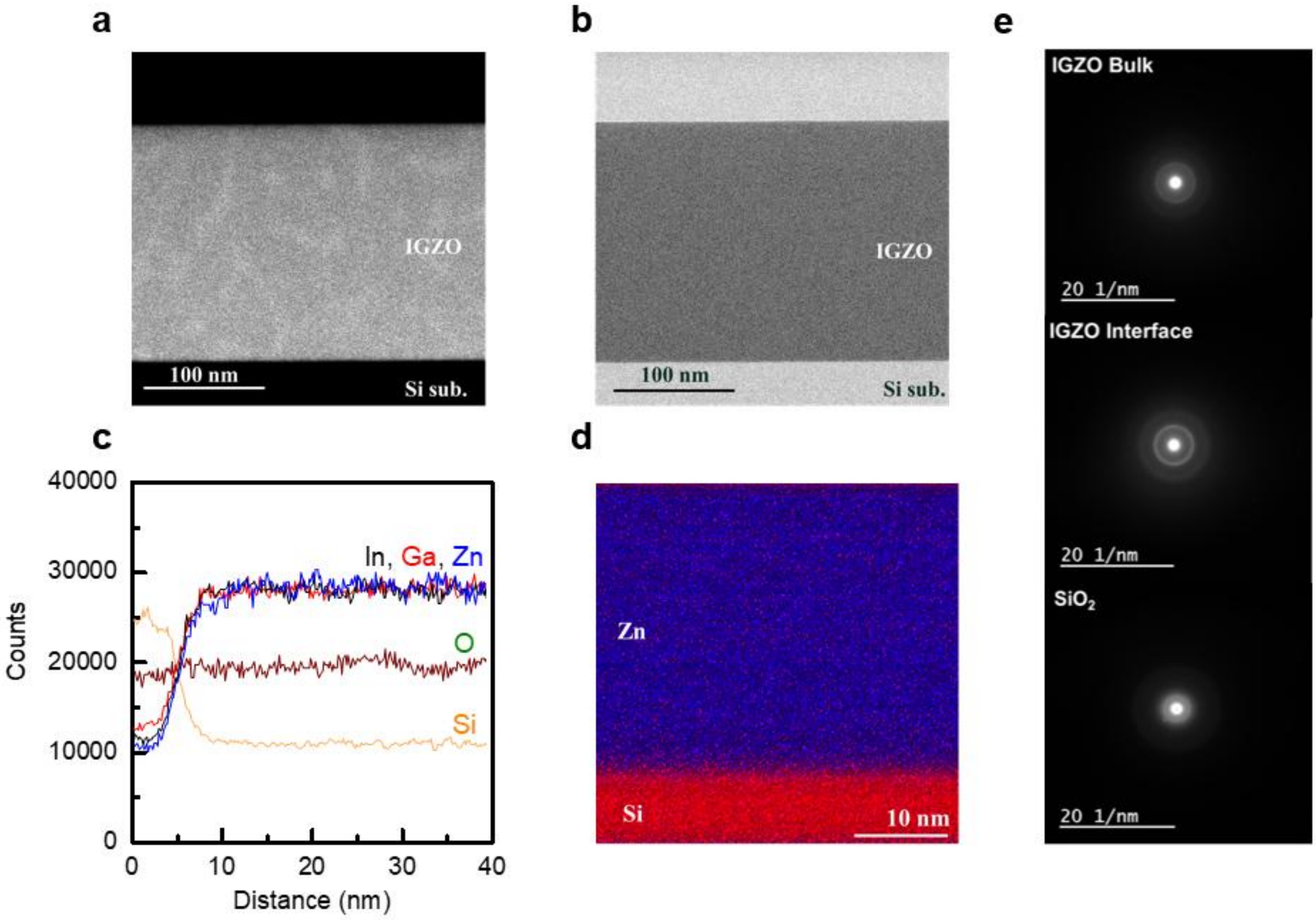

Figure S2. Cross-sectional STEM and EDS analyses of a-IGZO111. (a) HAADF-STEM and (b) bright-field STEM images of the a-IGZO111 film on a SiO_2/Si substrate. The mottled contrast in (a) originates from the carbon support mesh used for TEM specimen mounting and is not attributed to the a-IGZO microstructure. Neither image shows distinct contrast associated with crystallization or phase separation. (c) EDS line profiles of In, Ga, Zn, O, and Si across the a-IGZO111/SiO_2 interface. (d) EDS elemental maps of Zn and Si. (e) Electron diffraction patterns obtained from the a-IGZO bulk, the near-interface region, and the SiO_2 layer. The diffuse-ring spacing decreases from 3.1 Å in the bulk region to 3.0 Å in the near-interface region.

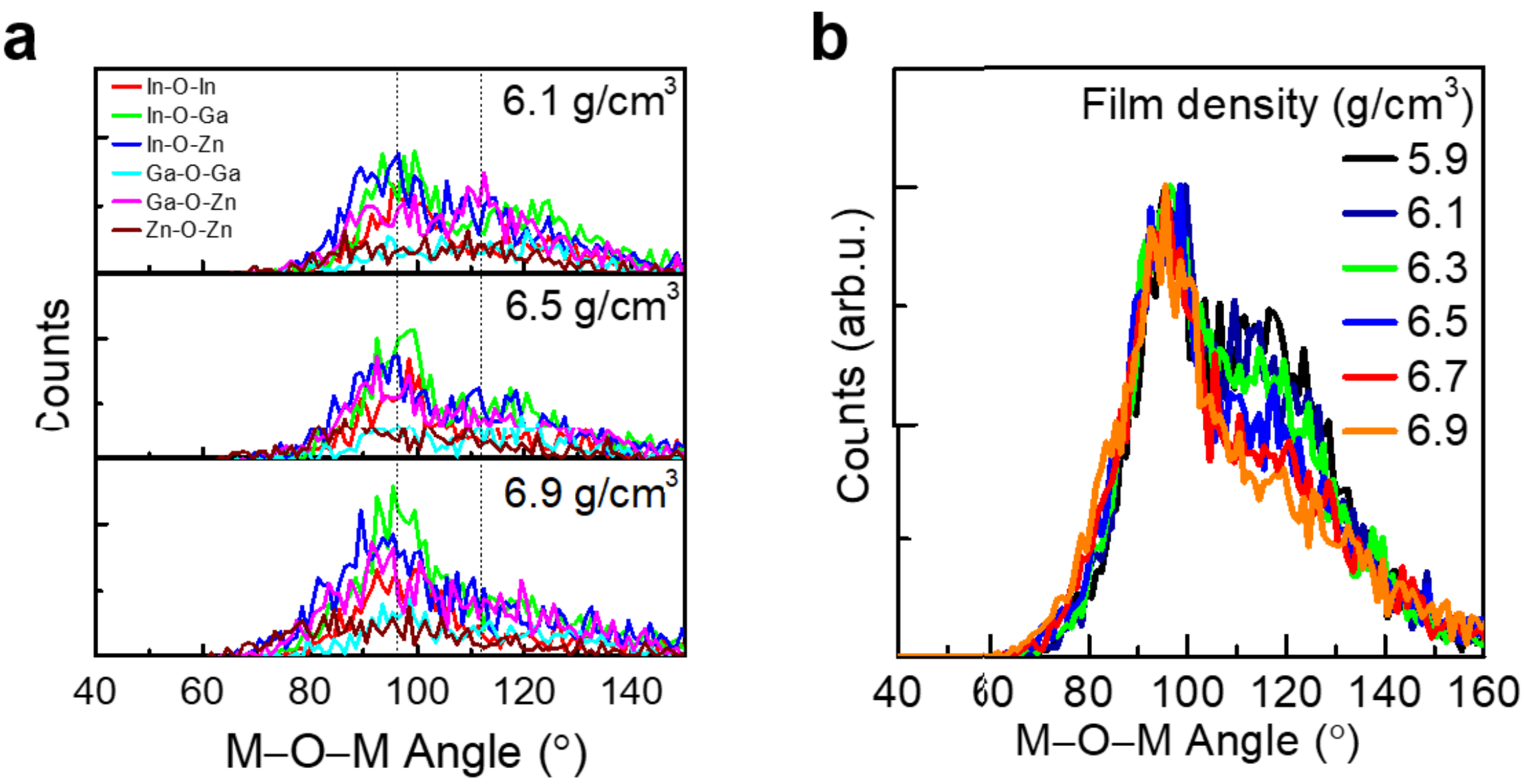

Figure S3. AIMD analysis of the *M*–O–*M* bond-angle distribution as a function of film density. (a) Bond-angle distributions decomposed into the individual *M*–O–*M* connections (In–O–In, In–O–Ga, In–O–Zn, Ga–O–Ga, Ga–O–Zn, and Zn–O–Zn) at film densities of 6.1, 6.5, and 6.9 g/cm^3. (b) Total *M*–O–*M* bond-angle distribution, summed over all bonding components, for film densities from 5.9 to 6.9 g/cm^3. As the density increases, the low-angle component increases, whereas the higher-angle component decreases.

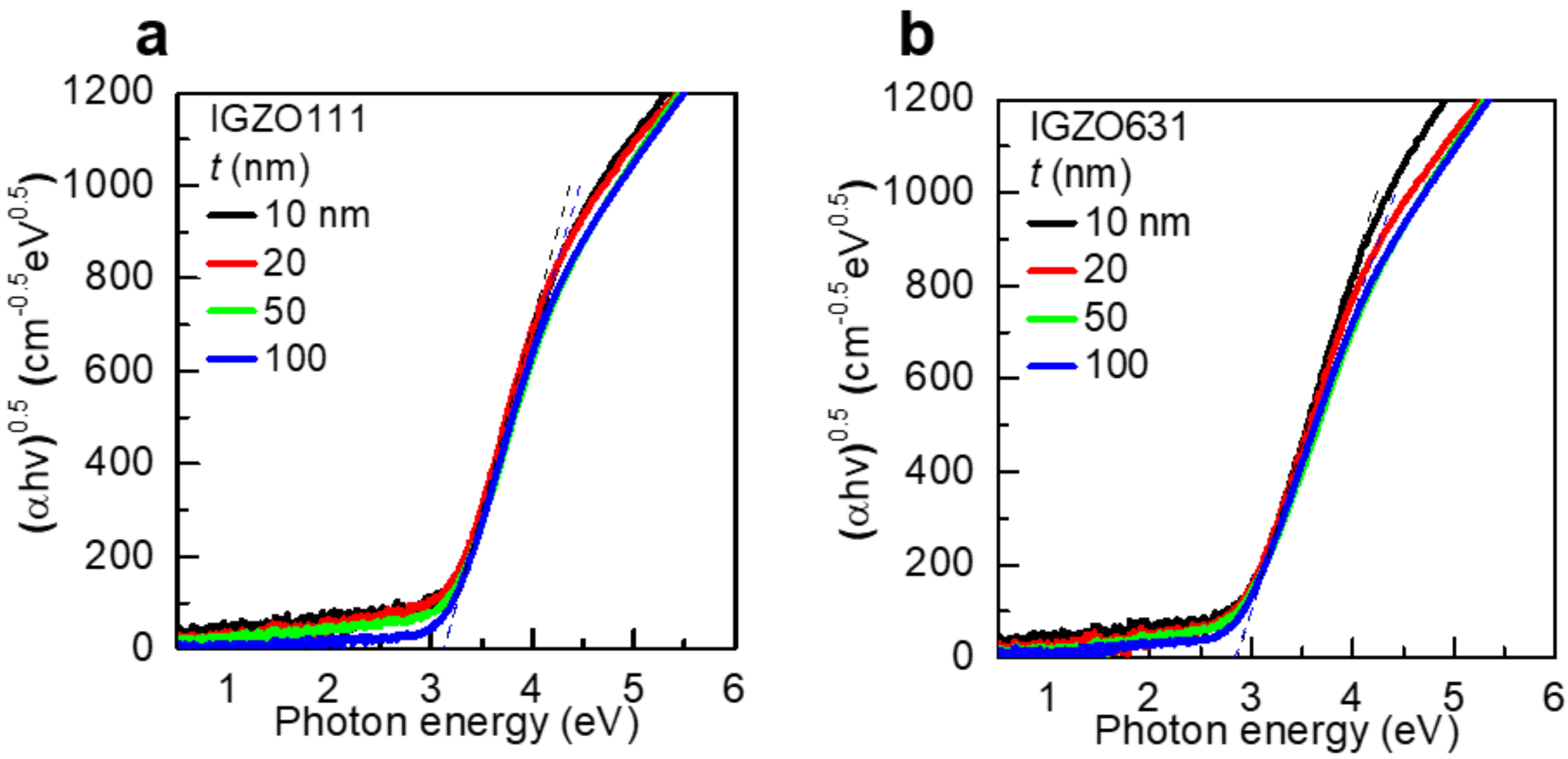

Figure S4. Tauc plots for (a) IGZO111 and (b) IGZO631 at thicknesses of 10, 20, 50, and 100 nm. The dashed lines indicate the linear extrapolation used to estimate the optical gap. For both compositions, the extracted optical gaps show no measurable change down to 10 nm.

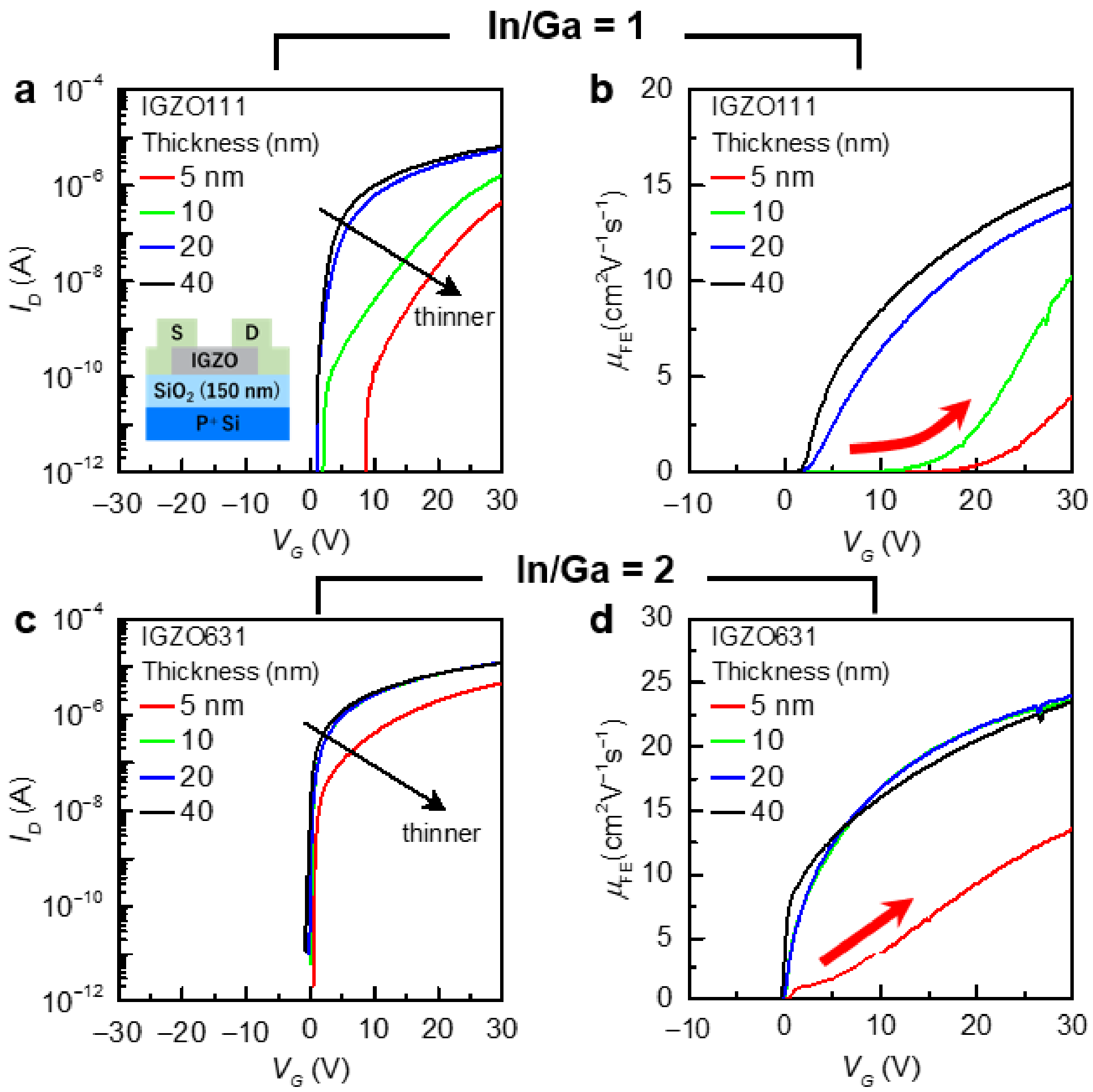

Figure S5. Complete transfer characteristics and field-effect mobility (μ_{FE}) curves for (a, b) IGZO111 (In/Ga = 1) and (c, d) IGZO631 (In/Ga = 2) at t_{ch} = 5, 10, 20, and 40 nm. The inset in (a) shows the bottom-gate device structure.

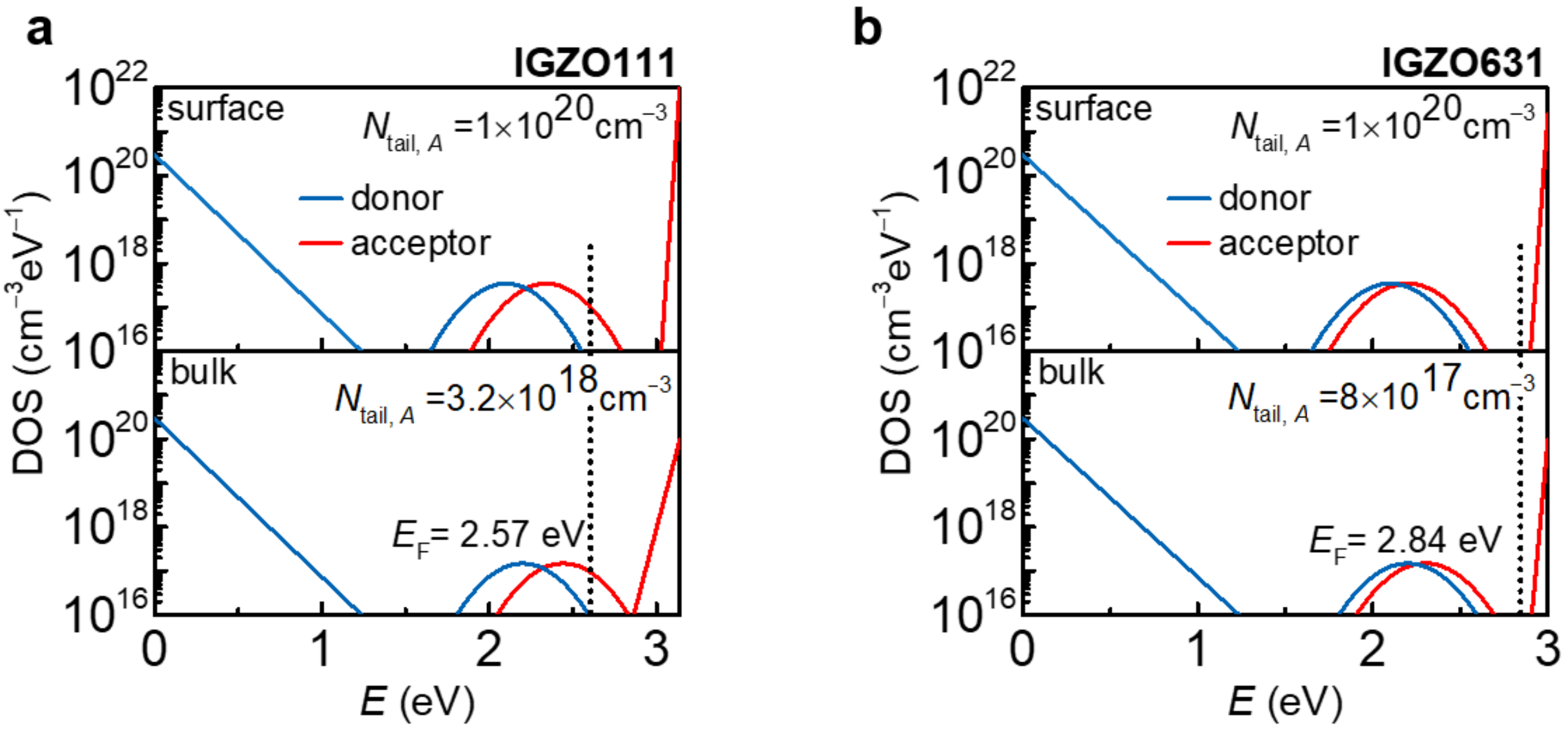

Figure S6. Complete TCAD-extracted DOS distributions for (a) IGZO111 and (b) IGZO631, including donor- and acceptor-like tail and Gaussian states in the surface and bulk regions. The dashed lines indicate the equilibrium E_F values calculated assuming carrier concentrations of 1×10^{17} cm^{-3} for IGZO631 and 1×10^{16} cm^{-3} for IGZO111, reflecting their In-rich and Ga-rich compositions, respectively.

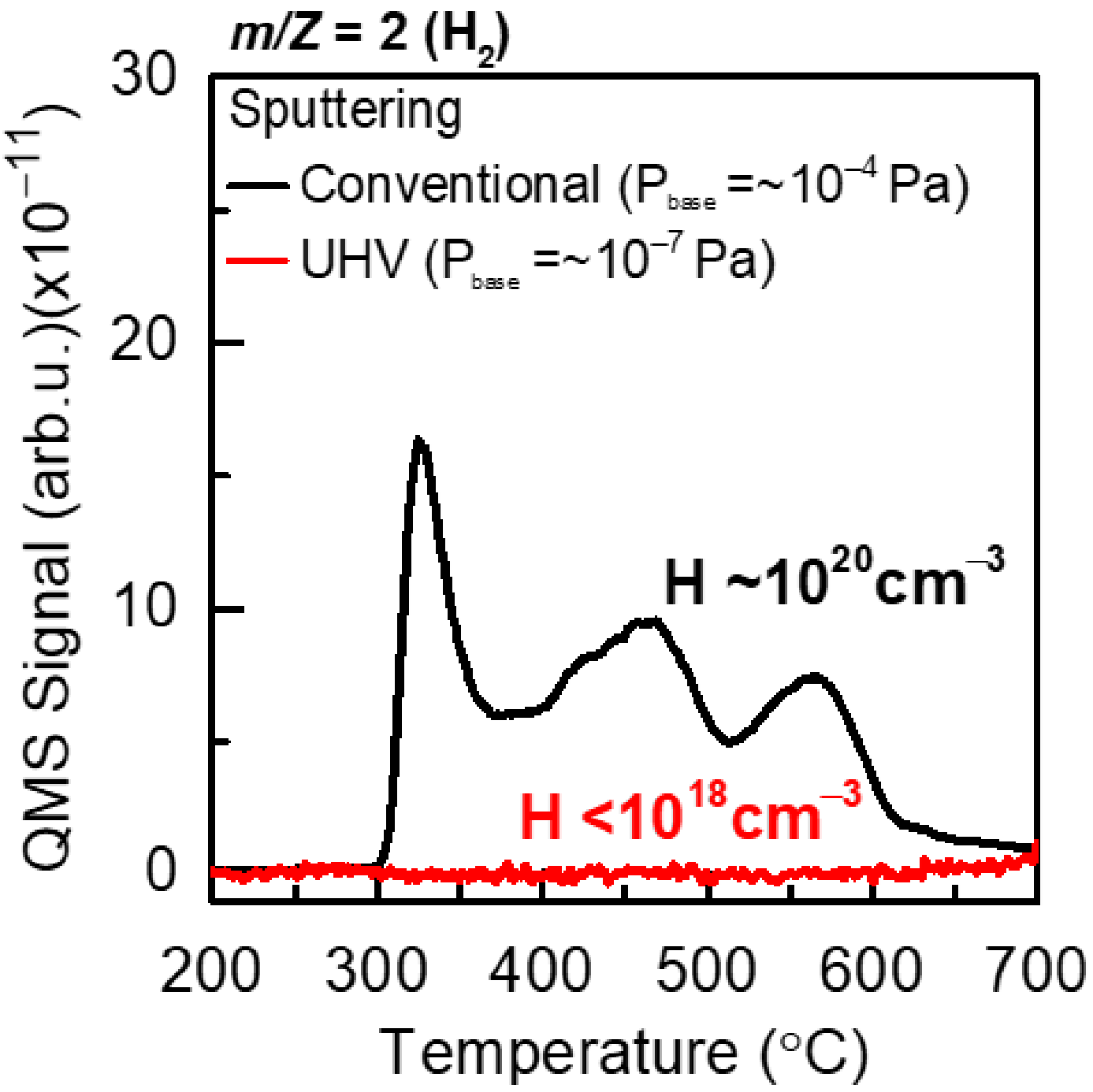

Figure S7. Thermal desorption spectroscopy (TDS) spectra of a-IGZO films deposited under conventional and UHV sputtering conditions. No H_2 desorption signal was detected from the UHV-sputtered film, indicating that the hydrogen concentration was below the detection limit of approximately 10^{18} cm^{-3}.

Table S1. Electron probe microanalysis (EPMA) of the cation composition of a-IGZO111 films as a function of thickness. Values are reported as the average ± standard deviation from 16-point grid measurements.

Film thickness (nm)	In (at%)	Ga (at%)	Zn (at%)
5	35.5±2.2	31.7 ± 2.1	32.8 ± 2.3
10	35.5±1.2	32.3 ± 1.1	32.2 ± 0.8
20	35.8±0.8	32.1 ± 0.6	32.1 ± 0.4
40	35.9±0.4	32.0 ± 0.4	32.1 ± 0.2
80	35.9±0.3	31.9 ± 0.4	32.2 ± 0.2

Table S2. Device-to-device variation in V_{th}, *SS*, and μ_{FE} for IGZO111 and IGZO631 TFTs with channel thicknesses of 5 and 40 nm. Each value represents the mean ± standard deviation obtained from five TFTs with the same W/L fabricated on the same sample.

Composition	t_{ch} (nm)	V_{th} (V)	*SS* (V/dec)	μ_{FE} (cm^2/V·s)
IGZO111	5	10.5 ± 1.1	1.12 ± 0.28	4.5 ± 0.5
IGZO111	40	1.5 ± 0.1	0.17 ± 0.07	15.1 ± 0.1
IGZO631	5	0.5 ± 0.2	0.14 ± 0.03	13.8 ± 0.5
IGZO631	40	−0.2 ± 0.2	0.09 ± 0.02	23.7 ± 0.4

Table S3. Density-of-states (DOS) parameters used in the device simulations for IGZO111 and IGZO631, extracted separately for the surface and bulk regions. g_{TA}, density of acceptor-like tail states at $E = E_C$; W_{TA}, characteristic slope of acceptor-like tail states; g_{GA}, peak density of acceptor-like Gaussian states; E_{GA}, peak energy of acceptor-like Gaussian states; W_{GA}, width of acceptor-like Gaussian states; g_{TD}, density of donor-like tail states at $E = E_V$; W_{TD}, characteristic slope of donor-like tail states; g_{GD}, peak density of donor-like Gaussian states; E_{GD}, peak energy of donor-like Gaussian states (measured from the valence band); W_{GD}, width of donor-like Gaussian states.

DOS Parameters	IGZO111		IGZO631	
	Surface region	Bulk region	Surface region	Bulk region
g_{TA} (cm^{-3} eV^{-1})	1.25×10^{22}	1×10^{20}	1.25×10^{22}	1×10^{20}
W_{TA} (eV)	0.008	0.032	0.008	0.008
g_{GA} (cm^{-3} eV^{-1})	3.5×10^{17}	1.5×10^{17}	3.5×10^{17}	1.5×10^{17}
E_{GA} (eV)	0.8	0.7	0.8	0.7
W_{GA} (eV)	0.24	0.24	0.24	0.24
g_{TD} (cm^{-3} eV^{-1})	3.0×10^{20}	3.0×10^{20}	3.0×10^{20}	3.0×10^{20}
W_{TD} (eV)	0.12	0.12	0.12	0.12
g_{GD} (cm^{-3} eV^{-1})	3.5×10^{17}	1.5×10^{17}	3.5×10^{17}	1.5×10^{17}
E_{GD} (eV)	2.1	2.2	2.1	2.2
W_{GD} (eV)	0.24	0.24	0.24	0.24

Table S4. CB tail width (W_{TA}) and integrated tail-state density (N_{TA}) for IGZO111 and IGZO631 in the surface and bulk regions. To avoid overparameterization, only the bulk W_{TA} was independently varied between the two compositions. The corresponding integrated tail-state density was calculated as $N_{TA} = g_{TA} \times W_{TA}$ from the exponential tail-state distribution, while all other parameters were identical.

Composition	Surface region		Bulk region	
	W_{TA} (meV)	N_{TA} (cm^{-3})	W_{TA} (meV)	N_{TA} (cm^{-3})
IGZO111	8	1×10^{20}	32	3.2×10^{18}
IGZO631	8	1×10^{20}	8	8×10^{17}
